# Karyopherin α-2 Mediates MDC1 Nuclear Import through a Functional Nuclear Localization Signal in the tBRCT Domain of MDC1

**DOI:** 10.3390/ijms21072650

**Published:** 2020-04-10

**Authors:** Kamalakannan Radhakrishnan, Seon-Joo Park, Seok Won Kim, Gurusamy Hariharasudhan, Seo-Yeon Jeong, In Youb Chang, Jung-Hee Lee

**Affiliations:** 1 Laboratory of Genomic Instability and Cancer therapeutics, Cancer Mutation Research Center, Chosun University, Gwangju 61452, Korea; kmknphd@gmail.com (K.R.); parksj@chosun.ac.kr (S.-J.P.); chosunns@chosun.ac.kr (S.W.K.); haribiochem08@gmail.com (G.H.); jsyshy@naver.com (S.-Y.J.); 2 Department of Premedical Sciences, College of Medicine, Chosun University, Gwangju 61452, Korea; 3 Department of Neurosurgery, College of Medicine, Chosun University, Gwangju 61452, Korea; 4 Department of Cellular and Molecular Medicine, College of Medicine, Chosun University, Gwangju 61452, Korea; 5 Department of Anatomy, College of Medicine, Chosun University, Gwangju 61452, Korea; iyjang@chosun.ac.kr

**Keywords:** MDC1, KPNA2, DNA damage response, homologous recombination repair, nuclear import

## Abstract

Mediator of DNA damage checkpoint protein 1 (MDC1) plays a vital role in DNA damage response (DDR) by coordinating the repair of double strand breaks (DSBs). Here, we identified a novel interaction between MDC1 and karyopherin α-2 (KPNA2), a nucleocytoplasmic transport adaptor, and showed that KPNA2 is necessary for MDC1 nuclear import. Thereafter, we identified a functional nuclear localization signal (NLS) between amino acid residues 1989–1994 of the two Breast Cancer 1 (BRCA1) carboxyl-terminal (tBRCT) domain of MDC1 and demonstrated disruption of this NLS impaired interaction between MDC1 and KPNA2 and reduced nuclear localization of MDC1. In KPNA2-depleted cells, the recruitment of MDC1, along with the downstream signaling p roteins Ring Finger Protein 8 (RNF8), 53BP1-binding protein 1 (53BP1), BRCA1, and Ring Finger Protein 168 (RNF168), to DNA damage sites was abolished. Additionally, KPNA2-depleted cells had a decreased rate of homologous recombination (HR) repair. Our data suggest that KPNA2-mediated MDC1 nuclear import is important for DDR signaling and DSB repair.

## 1. Introduction

Mediator of DNA damage checkpoint protein 1 (MDC1) plays a vital role in double stranded breaks (DSBs) repair through the DNA damage response (DDR) signal transduction pathway. It acts as a mediator of the DDR pathway by facilitating the recruitment of additional repair proteins to DNA damage sites [1,2,3]. MDC1 protein contains a forkhead-associated (FHA) domain at the N-terminus and two BRCA1 carboxyl-terminal (tBRCT) domains at the C-terminus. The central region consists of 14 repeated sequences of approximately 41 amino acids each that makes up the DNA-PKcs/Ku binding region [4]. In the event of DNA damage, MDC1 is hyper-phosphorylated in an Ataxia-Telangiectasia Mutated (ATM)-dependent manner and re-localized to the damaged region. It then interacts with phosphorylated γ-H2AX via the tBRCT domain and recruits other DDR proteins [5,6,7,8]. MDC1 is also required for events that take place subsequent to the recruitment of repair proteins, including phosphorylation and activation of the repair proteins [9]. Homologous recombination (HR) functions predominantly when homologous sister chromatids occur during S/G2 phase and eliminate DSBs in an error-free manner [10]. In addition to its major role as a platform for DDR, MDC1 also plays primordial roles in HR repair via promoting recruitment of Rad51, which is a key player of HR, to DBS sites [11]. Any delay or impairment in the recruitment of MDC1 to the nucleus leads to overall deficient DDR signal transduction.

Karyopherin α-2 (KPNA2), also known as importin α-1, is one of seven members of the karyopherin/importin-α family. It contains an N-terminal hydrophilic domain which binds to importin-β, a central hydrophobic region, and a short acidic C-terminus. The central hydrophobic region is comprised of 10 armadillo (ARM) repeats, which bind to the nuclear localization sequence (NLS) of cargo proteins and transport them to the nucleus through a nuclear pore complex (NPC) by forming a heterodimeric complex with importin-β [12,13,14]. This nuclear transport process takes place in two steps: firstly, energy-independent docking of cargo proteins to the nuclear membrane, and secondly, energy-dependent transport of cargo proteins through the NPC [15].

In this study, we identified KPNA2 as a specific nuclear import adaptor for MDC1 and demonstrated that the nuclear localization of MDC1 is partly regulated by NLS motif located between residues 1989–1994 of the tBRCT domain. We then showed that depletion of KPNA2 decreased HR repair and impaired recruitment of MDC1, as well as its downstream signaling proteins to DNA damage sites. These findings establish that KPNA2 regulates MDC1-mediated DDR by promoting MDC1 nuclear import.

## 2. Results

### 2.1. KPNA2 Binds to the tBRCT Domain of MDC1

**Figure 1:**
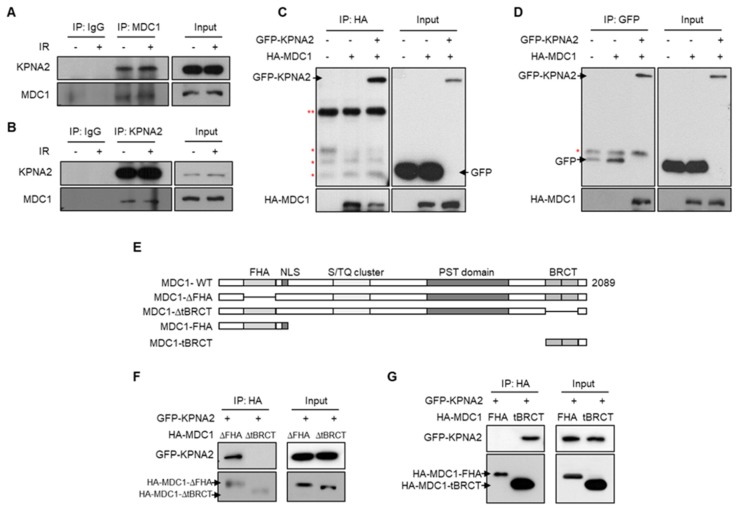
KPNA2 interacts with two BRCA1 carboxyl-terminal (tBRCT) domain of mediator of DNA damage checkpoint protein 1 (MDC1). (**A**,**B**) HeLa cells, with or without exposure to ionizing radiation (IR), and whole-cell lysates were subjected to immunoprecipitation using an anti-MDC1 (**A**) or anti-KPNA2 (**B**) antibodies, followed by Western blotting using antibodies against either KPNA2 or MDC1. (**C**,**D**) HA-MDC1 and GFP-KPNA2 were co-transfected into HEK293T cells and exposed to IR. Whole cell lysates were subjected to immunoprecipitation using anti-HA (**C**) or anti-GFP (**D**) antibodies, followed by Western blotting using antibodies against either HA or GFP. *, nonspecific band. **, IgG heavy chain band. (**E**) Schematic representation of domain structure of wild-type MDC1 and the various deletion mutations is shown. (**F**,**G**) GFP-KPNA2 were transfected into HEK293T cells along with indicated deletion mutants of MDC1 (**F**) or HA-MDC1-FHA and HA-MDC1-tBRCT (**G**). Cell lysates were immunoprecipitated using anti-HA antibody and analyzed by Western blotting using the indicated antibodies.

To explore the molecular mechanism by which MDC1 regulates DDR signaling, we performed a yeast two-hybrid screen using the C-terminal fragment (amino acids 1882–2089) of human MDC1 as the bait and looked for novel protein interactions. One of the positive clones isolated from the 2 × 10^6^ transformants was a nuclear transporter, KPNA2 [1]. To determine whether the interaction between MDC1 and KPNA2 was relevant in the context of a cell, we performed immunoprecipitation assays. HeLa cells were treated with Ionizing Radiation (IR) to induce DSBs, and then endogenous MDC1 was immunoprecipitated using an MDC1-specific antibody. Immunoprecipitates were analyzed using Western blotting with either anti-KPNA2 or anti-MDC1 antibodies. Analysis with anti-MDC1 antibody showed an interaction between MDC1 and KPNA2, and treatment with IR did not significantly change the amount of MDC1 that bound to KPNA2 (Figure 1A). A reciprocal assay using anti-KPNA2 antibody confirmed this interaction (Figure 1B). When normal rabbit IgG was used as a negative control, neither MDC1 nor KPNA2 precipitated, indicating that the interaction between MDC1 and KPNA2 was specific. This result with endogenous proteins was corroborated using exogenous proteins in a reciprocal co-immunoprecipitation assay using Hemagglutinin (HA)-tagged MDC1 and Green fluorescent protein (GFP)-tagged KPNA2 (Figure 1C,D). These results support the prediction that there is an intracellular interaction between MDC1 and KPNA2.

Furthermore, we investigated whether KPNA2 binds to the tBRCT domain of MDC1 since the C-terminal fragment (amino acids 1882–2089) of human MDC1 contains a tBRCT domain, which is essential for MDC1 recruitment to DNA damage sites. To this end, we expressed GFP-KPNA2, along with HA-tBRCT-deleted MDC1 (ΔtBRCT) or HA-FHA-deleted MDC1 (ΔFHA), in HEK293T cells (Figure 1E) and attempted to immunoprecipitate MDC1 using anti-HA antibody. We found that ΔFHA-MDC1 co-immunoprecipitated with GFP-KPNA2, but ΔtBRCT-MDC1 did not (Figure 1F). To confirm that the tBRCT domain of MDC1 is indeed important for the binding of MDC1 and KNPA2, GFP-KPNA2 was expressed in HEK293T cells, together with the FHA or tBRCT domain of MDC1, and co-immunoprecipitation experiments were performed using anti-HA antibody. HA-tBRCT, but not HA-FHA, co-immunoprecipitated with GFP-KPNA2 (Figure 1G), indicating that the tBRCT domain of MDC1 is required for binding to KPNA2.

### 2.2. KPNA2 Knockdown Results in Impaired Nuclear Translocation of MDC1

**Figure 2:**
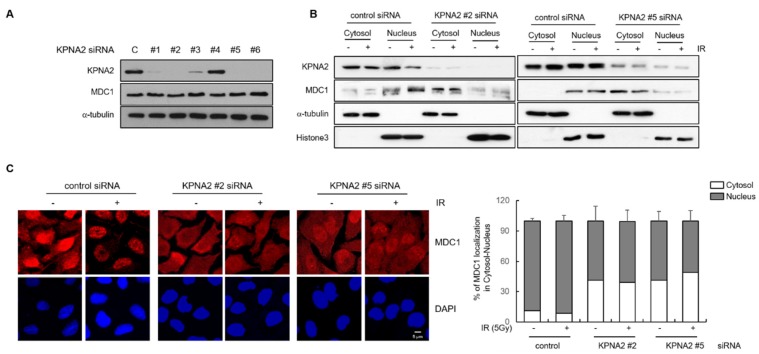
Impaired nuclear translocation of MDC1 in KPNA2-depleted cells. (**A**) Five siRNAs (KPNA2 siRNA #1 ~ KPNA2 siRNA #5), each designed against different region of the KPNA2 target gene, were transiently transfected into HeLa cells. Western blotting was carried out to determine expression levels of KPNA2 and MDC1 in control or KPNA2-depleted cells. α-tubulin was included as an internal control. (**B**) Control or KPNA2-depleted HeLa cells were irradiated with or without IR, and the cytosol and nuclear fractions were isolated. The cytosol and nuclear extracts were subjected to Western blotting using antibodies against MDC1 and KPNA2. Histone H3 and α-tubulin were used as positive controls for nuclear and cytoplasmic fraction, respectively. (**C**) Same cells, as described in (**B**), were fixed for immunofluorescence staining (IF) of MDC1. DAPI (4′,6-diamidino-2-phenylindole) was used for nuclear staining (left). Scale bar; 5 μm. Bars graph obtained from IF of control and KPNA2 siRNA transfected cells showing the percentage in different cellular localization (right).

Because KPNA2 binds to MDC1 and is a known nuclear transport protein, we examined its role in the translocation of MDC1 from the cytosol to the nucleus when repair of IR-induced DSBs was necessitated. To test this, we depleted KPNA2 from HeLa cells using five different siRNAs and found that the expression of KPNA2 was reduced by more than 90% in HeLa cells transfected with KPNA2 siRNA #2 or #5 (Figure 2A). Most of our experiments were performed using KPNA2 siRNA #2 and #5, and, in some experiments, KPNA2 siRNA #2 were simultaneously used. To determine whether or not the KPNA2 knockdown impaired nuclear transport of MDC1, after control and KPNA2 knockdown cells had been irradiated with IR, the amount of MDC1 protein in cytosolic and nuclear extracts was determined using Western blotting. The presence of histone 3 and α-tubulin, a nuclear- and cytosol- specific protein, respectively, confirmed the specificity of the nuclear and cytosol fraction. In control cells, the amount of MDC1 was mainly presented in the nuclear fraction either before or after irradiation (Figure 2B). However, in KPNA2 knockdown cells, the large amount of MDC1 protein was in the cytosol. Since there was no difference in the levels of endogenous MDC1 expression between control cells and KPNA2-deficient cells (Figure 2A), these results indicated that, without KPNA2, MDC1 was not efficiently translocated to the nucleus. Additionally, immunofluorescent staining revealed that in the control cells, MDC1 was mainly stained in the nucleus, but, in the KPNA2-deficient cells, it was mainly stained in the cytoplasm (Figure 2C). Collectively, these results indicate that KPNA2, a nuclear transporter protein, is required for MDC1 translocation from the cytosol to the nucleus.

### 2.3. Putative NLS of MDC1 is Critical for Its Binding to KPNA2 and Contributes to Its Nuclear Import

**Figure 3:**
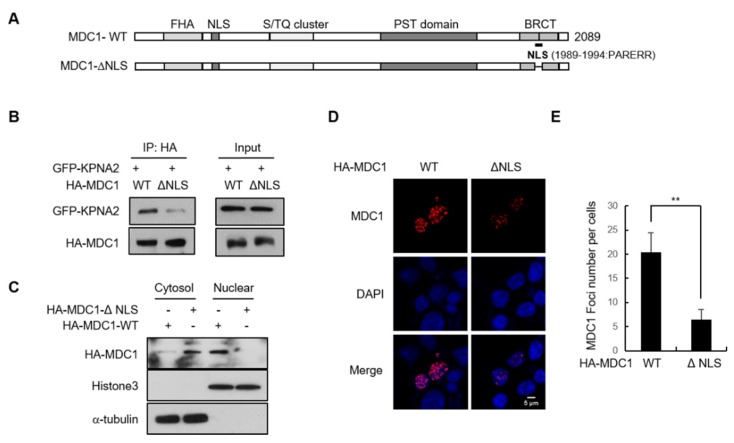
Identification of functional NLS in MDC1. (**A**) A schematic representation of HA-tagged wild-type MDC1 (MDC1-WT) and NLS-deleted HA-MDC1 (MDC1-ΔNLS). Putative nuclear localization signal (NLS, PARERR) is shown. (**B**) GFP-KPNA2 were transfected into HEK293T cells along with HA-MDC1-WT or HA-MDC1-ΔNLS. Whole cell lysates were then subjected to immunoprecipitation using anti-HA antibody, followed by Western blotting using indicated antibodies. (**C**) HeLa cells were transfected with MDC1-WT or MDC1-ΔNLS, and the cytosol and nuclear fractions were isolated. The cytosol and nuclear extracts were subjected to Western blotting using indicated antibodies. (**D**,**E**) MDC1-WT or MDC1-ΔNLS-transfected HeLa cells were irradiated with IR and the number of IR-induced HA-MDC1 foci was calculated. Representative images (**D**) and quantification (**E**) of HA-MDC1 foci are shown. Scale bar; 5 μm. Data represent mean ± SD (*n* = 3), ** *p* < 0.01.

KPNA2 binds to classic NLS motifs of cargo proteins and facilitates their nuclear import [16]. To analyze NLS prediction in the tBRCT domain, we used NLStradamus software [17], a simple hidden Markov model (HMM) for NLS prediction, and found that a putative NLS (PARERR) existed and was located in amino acids 1989–1994 of tBRCT domain (Figure 3A). To characterize this putative NLS of the tBRCT domain in MDC1, we tested whether this NLS of the tBRCT domain is responsible for the interaction between MDC1 and KNPA2 via co-immunoprecipitation assays. To this end, we generated putative NLS deletion mutant from wild-type HA-MDC1 (HA-MDC1-ΔNLS). We transfected HEK293T cells with GFP-KPNA2, along with HA-MDC1-WT (Wild type) or HA-MDC1-ΔNLS, and performed immunoprecipitation assay with anti-HA antibody. As expected, anti-HA antibody immunoprecipitated HA-MDC1-WT (Figure 3B). However, KPNA2 was weakly detected in the immunoprecipitating complex containing HA-MDC1-ΔNLS, suggesting that these amino acid residues are required for MDC1 to associate with KPNA2. To determine whether these amino acid residues of MDC1 are responsible for its nuclear import, control and KPNA2-depleted HeLa cells were transfected with wild-type HA-MDC1 or HA-MDC1-ΔNLS, followed by subcellular fractionation and analysis via immunoblotting or immunofluorescence detection of MDC1. Immunoblotting analysis revealed that transiently expressed HA-MDC1-ΔNLS was predominantly present in the cytoplasm (Figure 3C). Furthermore, quantitative fluorescence imaging of transfected cells revealed that the IR-induced nuclear foci of HA-MDC1-ΔNLS was significantly reduced compared to that of HA-MDC1 (Figure 3D,E). Collectively, these results identify the amino acid motif (PARERR) of MDC1 as a nuclear localization element and indicate that KPNA2 interacts with this element for MDC1 nuclear import.

### 2.4. KPNA2 Knockdown Results in Reduced IR-induced MDC1 Foci Formation and Impaired Homologous Recombination (HR) Repair

**Figure 4:**
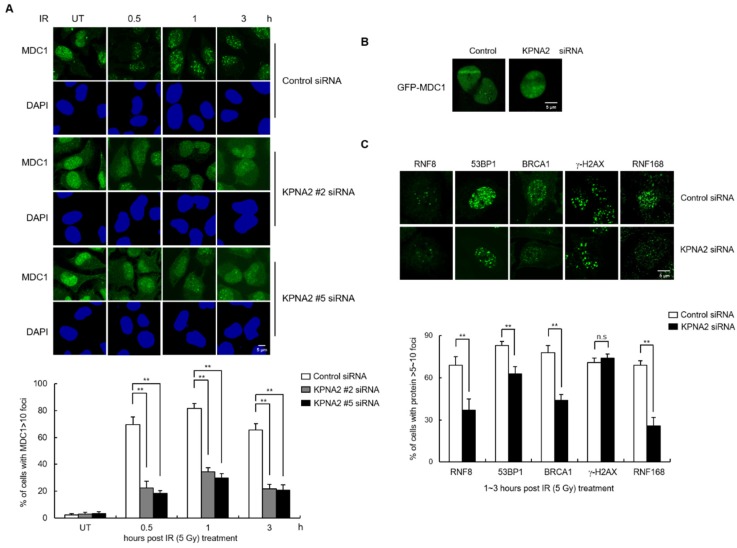
KPNA2 knockdown reduces IR-induced MDC1 foci formation. (**A**) Control and KPNA2-depleted HeLa cells were treated with or without exposure to 5 Gy of IR and fixed at the indicated time points. Immunostaining was performed using MDC1 antibody and Nuclei were stained DAPI (upper). The percent of cells containing >10 nuclear MDC1 foci was then calculated (lower). Scale bar, 5 μm. (**B**) Control and KPNA2-depleted cells were transfected with GFP-KPNA2 and laser-microirradiated using 405 UV laser. The representative images were shown after laser-microirradiation. Scale bar; 5 μm. (**C**) Control or KPNA2-depleted HeLa cells were exposed to 5 Gy of IR for 1~3 h and immunostained with indicated antibodies. The representative images (upper) and percentage (lower) of cells containing >5–10 nuclear RNF8, 53BP1, BRCA1, RNF168, or γ-H2AX foci were shown. Scale bar; 5 μm. Data are presented as means ± s.d. P value are based on two-tailed Student’s *t*-test: **p < 0.01 ns, not significant.

Because we observed that KPNA2 was involved in MDC nuclear import, we predicted that KPNA2 might regulate the DDR pathway by promoting MDC1 function. To verify this, control and KPNA2 knockdown cells were irradiated with IR and the number of MDC1 foci was measured at subsequent time points. We found that MDC1 foci formation was significantly reduced in KPNA2 deficient cells over 0.5, 1, and 3 h after irradiation compared to control cells (Figure 4A). Quantification of the data indicated that the percentage of KPNA2 knockdown cells containing more than 10 foci of MDC1 remained at only ~25–30%, whereas for control cells, this number had risen to ~75%. To further explore the effect of KPNA2 on the MDC1 foci formation, control and KPNA2 knockdown cells were transfected with GFP-tagged MDC1 and then laser-microirradiated with 405-nm laser to induced DSBs and allowed to recover for 5 min. Consistently, the accumulation of GFP-MDC1 at the site of laser microirradiation was markdedly reduced in KPNA2 depleted cells (Figure 4B). MDC1 is required for the retention of additional DDR proteins at DNA damage sites; thus, it is considered an upstream regulator of the DDR [4,18,19]. Therefore, we predicted that KPNA2 depletion would also affect nuclear localization of other DDR factors to DSB sites. To test this hypothesis, we monitored IR-induced nuclear foci formation for RNF8, 53BP1, BRCA1, γ-H2AX, and RNF168 in the presence or absence of KPNA2. Results indicated that depletion of KPNA2 resulted in fewer foci for four of these proteins, RNF8, 53BP1, BRCA1, and RNF168 (Figure 4C), similar to what had been previously observed for MDC1-deficient cells. On the other hand, γ-H2AX, a protein that acts upstream of MDC1 in the DDR pathway, was unaffected by the presence or absence of KPNA2. Together, these data suggest that KPNA2-mediated MDC1 nuclear translocation is particularly important for DNA damage signaling regulated by MDC1, since the recruitment of both MDC1 and its downstream proteins are critical for DDR.

**Figure 5:**
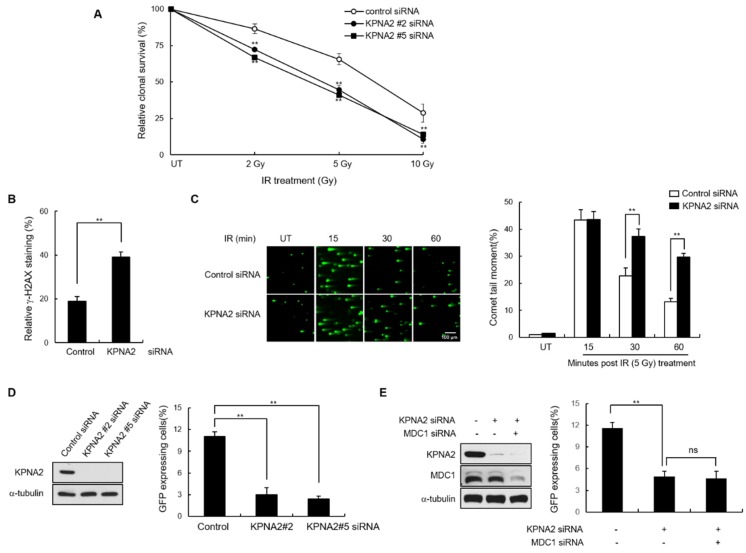
KPNA2 promotes HR repair. (**A**) Control and KPNA2-depleted HeLa cells were exposed to the indicated dose of IR and assessed for colony forming ability. The cell viability of untreated cells is defined as 100%. Data presented as mean ± s.d. (**B**) Control and KPNA2-depleted HeLa cells were exposed to IR. After 24 h, immunostaining was performed using γ-H2AX antibody. The percentage of cells containing >10 γ-H2AX foci was then calculated. Data are presented as means ± s.d. (**C**) Control and KPNA2-depleted HeLa cells were untreated or treated with 5 Gy γ-irradiation. At the indicated time points, cells were harvested to carry out comet assay under neutral condition. Comet images were captured using fluorescence microscopy, and comet tail moment was analyzed using Komet 5.5 analysis software. Representative comet images obtained at different time points are shown. Scale bar; 100 μm. Changes in the tail moments between control and KPNA2 knockdown cells after IR treatment are represented in histogram. Data presented as mean ± s.d. (**D**,**E**) DR-GFP-U2OS cells transfected with the indicated siRNA combinations. The level of endogenous KPNA2 and MDC1 were analyzed by Western blotting (left). The GFP-positive cells were measured by Fluorescence-activated cell sorting (FACS). Data represent mean ± s.d. (*n* = 3), ** *p* < 0.01. ns, not significant.

Because MDC1 directly mediates DSB repair through HR [11], and because KPNA2 regulates MDC1 nuclear translocation, we predicted that the HR of DSBs would be affected in KPNA2 knockdown cells. As previously reported, we observed hypersensitivity to IR and defective DNA repair in the absence of KPNA2 expression through clonal survival assay, late γ-H2AX staining and neutral comet assay, respectively (Figure 5A–C). To determine whether KPNA2 plays a role in regulating HR repair, DR-GFP-U2OS cells were transiently transfected with either control siRNA or two different KPNA2 siRNA. Immunoblotting confirmed that endogenous KPNA2 protein was significantly reduced, compared to control siRNA transfected cells. To evaluate HR efficiency, KPNA2-depleted DR-GFP-U2OS cells were infected with the I-SceI expressing adenovirus, and the GFP-positive cells were measured, followed by flow cytometry analysis (Figure 5D). In three independent experiments, the HR efficiency was significantly reduced in the KPNA siRNA relative to control siRNA-transfected cells. Knockdown of MDC1 did not additionally reduce of DSB repair in KPNA2-depleted cells (Figure 5E), suggesting that MDC1 and KPNA2 function in the same pathway for HR repair.

## 3. Discussion

In this study, we have evaluated the role of KPNA2 in the MDC1-mediated DDR signal and DSB repair pathway. The key findings of this study are as follows: firstly, KPNA2 regulates MDC1 nuclear transport through interaction of NLS sequences in the MDC1 tBRCT domain. Secondly, KPNA2 depletion impaired MDC1 nuclear transport, without affecting MDC1 expression level, leading to a marked decrease in the recruitment of MDC1 and its downstream proteins to DNA damage sites after irradiation and decreased HR-mediated DSB repair.

Accumulating data suggest that DDR proteins are transported to the nucleus via NLS-dependent interaction with importin [20,21,22,23,24]. Although MDC1 is a well-known large nuclear protein that requires an active nuclear import mechanism to participate in DNA repair [2,8,25], the mechanism of its nuclear translocation remained unknown to date. In this study, we identified KPNA2 as the specific member of the importin-α adaptor family that facilitates MDC1 recognition by the import machinery. KPNA2 belongs to a family of transporter proteins known to translocate NLS-containing cargo proteins from the cytoplasm into the nucleus [21,26,27]. Selectivity for specific KPNA2 adaptor is mediated by specific NLS sequences in combination with structural features of cargo [26]. We thus hypothesized that KPNA2 may recognize the nuclear targeting signal of MDC1. Using the NLStradamus program, we were able to identify a putative NLS in the tBRCT domain of MDC1. Our data showed that putative NLS (amino acid residues 1989–1994) was critical for binding of MDC1 to KPNA2 and also as concomitant targets of positive regulation for MDC1 nuclear import. The depletion of the NLS of MDC1 or KNPA2 knockdown led to a significant decrease in MDC1 protein in the nucleus, suggesting that KPNA2 is the transport adaptor for the MDC1 nuclear import. To the best of our knowledge, this is the first experiment to show how MDC1 is transported into the nucleus.

MDC1 is recruited to DSB sites and functions as an assembly platform to trigger the recruitment of additional DDR factors to DNA damage sites, including RNF8, RNF168, BRCA1, and 53BP1 [4,18,19]. Thus, we looked for the effect of KPNA2 depletion on foci formation and found that IR-induced MDC1 nuclear foci were significantly lower in KPNA2 knockdown cells compared to control cells. Moreover, the recruitment of RNF8, 53BP1, BRCA1, and RNF168 to DNA damage sites was also compromised in KPNA2 knockdown cells. In contrast, γ-H2AX, a protein that acts upstream of MDC1 in the DDR, was unaffected by the presence or absence of KPNA2. These findings demonstrate that KPNA2 regulates DDR via MDC1 nuclear import.

HR is a repair pathway thought to be error-free because it uses undamaged sister chromatids as the template for repair [28]. As MDC1 plays an important role in HR [11], we also looked for the role of KPNA2 in HR. An assay for HR-mediated DNA repair using I-SceI-induced DSBs showed significantly lower rates of HR in KPNA2-depleted cells compared to control cells. Notably, depletion of both KPNA2 and MDC1 did not further decrease HR activity as observed with MDC1 depletion alone. Moreover, KPNA2 is known to be involved in the nuclear translocation of Nijmegen breakage syndrome 1 (NBS1 [20], a key regulator of the MRE11-RAD50-NBS1 (MRN) complex, which plays an important role in the DSB repair pathway. Thus, the role of KPNA2 in HR repair may be to promote the nuclear import of both MDC1 and NBS1.

Human importin-α family is classified into three subfamilies on the basis of their sequence homology: the α1 subfamily includes KPNA1, KPNA5, and KPNA6; the α2 subfamily includes KPNA2 and KPNA7; the α3 subfamily includes KPNA4 and KPNA3 [29]. The recently identified KPNA7 belongs to the same subfamily as KPNA2 and has a high amino acid similarity to KPNA2 [30,31]. Thus, KPNA7 may exhibit functional redundancy with KPNA2 regarding transport of MDC1 to the nucleus. However, a striking feature of KPNA7 is its localization to the nucleus under steady state conditions, while KPNA2 is predominantly cytoplasmic [30]. The difference in the intracellular distribution of these two proteins suggest that nucleocytoplasmic shuttling of KPNA7 differs from that of KPNA2. Importin-α isoforms contribute to distinctive physiological roles due to differential expression in tissues and differential binding affinity for these isoforms with NLS [32,33]. Nevertheless, it does not exclude the possibility that other subfamily proteins, including α1 and α3, are involved in the nuclear transfer of MDC1, which would be good topics for future study.

In conclusion, this study establishes the role of KPNA2 as a novel regulator of the DDR function of MDC1, as it controls MDC1 nuclear import. Furthermore, we demonstrated that nuclear import of MDC1 is controlled by its NLS interacting with KPNA2, which is essential for the MDC1-mediated DDR pathway. Our work provides evidence for a novel connection between KPNA2 and MDC1, and evidence for the interaction of KPNA2 and MDC1 as a mechanism for regulating the response of MDC1 to DNA damage.

## 4. Materials and Methods

### 4.1. Cell Culture

The human cervix carcinoma HeLa cells, human embryonic kidney HEK293T cells, and DR-GFP U2OS cells were cultured in Dulbecco’s modified Eagle’s medium (DMEM) supplemented with 10% heat-inactivated fetal bovine serum, 100 units/mL penicillin, and 100 mg/mL streptomycin sulfate (Invitrogen, Carlsbad, CA, USA). All cells were maintained in a humidified incubator containing 5% CO_2_ at 37 °C. Upon reaching 70–80% confluency, cells were digested with 0.5% trypsin-EDTA before being passaged. Cells in exponential growth were harvested for subsequent experiments. To induce DNA double strand breaks, exponentially growing cells were irradiated at 5 Gy from ^137^Cs source (Gammacell 3000 Elan irradiator, Best Theratronics, Ottawa, Canada) and allowed to recover at 37 °C incubator for various times.

### 4.2. Transfection of Small-Interference RNA (siRNA)

The five siRNA sequences against KPNA2 (NM_002266.2) are as follows: KPNA2 siRNA#1, 5’-GCAGCUAAGAAAGUACAUAdTdT-3′; KPNA2 siRNA #2, 5′-GCAUCAUGAUGAUCC AGAA dTdT-3′; KPNA2 siRNA #3, 5′-ACGAAUUGGCAUGGUGGUGAAdTdT-3′; KPNA2 siRNA #4, 5′-CCGGGUGUUGAUUCCGAAdTdT-3′; KPNA2 siRNA #5, 5′-CAGAUACC UG CUGGGCUAUUUCCU AdTdT-3′; MDC1 siRNA, 5′-UCCAGUGAAUCCUUGAGGUdTdT-3′; Negative control siRNA (Bioneer, Daejeon, Korea), 5′-CCUACGCCACCAAUUUCGUdTdT-3′. The control or KPNA2 siRNA were transiently transfected into the cells using Lipofectamine RNAiMAX (Invitrogen) according to the manufacturer’s instructions. At 48 h post transfection, the inhibition of KPNA2 or MDC1 was judged by western blot analysis.

### 4.3. Preparation of Plasmid Construction

The plasmids encoding with type, ΔFHA, ΔtBRCT-MDC1 were obtained from Zhenkun Lou and HA-FHA and HA-tBRCT-MDC1 construct were prepared by subcloning previously in our lab [1]. Full-length human KPNA2 cDNA was amplified from HeLa cDNA, and the PCR products were cloned into pEGFP-N3 vector. To construct the expression vector encoding MDC1-ΔNLS (PARERR), we performed mutagenesis using GENEART® Site-Directed Mutagenesis System (Invitrogen) according to the manufacturer’s instructions. The all constructs were confirmed by automated DNA sequencing.

### 4.4. Western Blot Analysis

For total cell lysates extraction, cells were lysed in ice-cold RIPA buffer (50 mM Tris-HCl (pH 7.5), 150 mM NaCl, 1% Nonidet P-40, 0.5% sodium deoxycholate, 0.1% sodium dodecyl sulfate, 1 mM dithiothreitol, 1 mM phenylmethanesulfonyl fluoride, 10 μg/mL leupeptin and 10 μg/mL aprotinin) for 30 min on ice. The supernatants were collected by centrifugation at 13,200 rpm for 20 min at 4 °C and then quantified using Bio-Rad protein assay (Bio-Rad Laboratories Inc., Hercules, CA USA). For cellular fractionation, cells were harvested and lyzed in cytosol extraction buffer (CEB; 10 mM HEPES pH 7.5, 3 mM MgCl_2_, 14 mM KCl, 5% glycerol, 1mM dithiothreitol, 1mM phenylmethanesulfonyl fluoride, 10 μg/mL leupeptin and 10 μg/mL aprotinin) for 10 min on ice. For complete lysis, 0.2% NP-40 was added followed by vortexing for 10 s. After centrifugation at 86,000× *g* for 2 min, the supernatant which is cytosolic fraction, was transferred to a new tube. The pellet was washed three times in CEB and then lysed in nuclear extraction buffer (NEB; 10 mM HEPES, 3 mM MgCl_2_, 400 mM NaCl, 5% glycerol, 1mM DTT, 1mM PMSF, 10 μg/mL leupeptin, and 10 μg/mL aprotinin) for 30 min at 4 °C, followed by centrifugation at 13,200 rpm for 30 min. The supernatant is nuclear fraction. Equal amounts of protein were separated by 6–12% SDS-PAGE, followed by electrotransfer onto a polyvinylidene difluoride membrane (PALL life sciences). The membranes were blocked for 1 h with TBS-t (10 mM Tris-HCl (pH 7.4), 150 mM NaCl and 0.1% Tween-20) containing 5% nonfat milk and then incubated with indicated primary antibodies overnight at 4 °C. The blots were washed four times for 15 min with TBS-t and then incubated for 1 h with peroxidase-conjugated secondary antibodies (1:5000, Jackson ImmunoResearch Inc, West Grove, PA, USA). The blots were washed four more times with TBS-t and developed using an enhanced chemiluminescence detection system (ECL; intron).

### 4.5. Immunoprecipitation

The protein extracts were precleared with protein A-Sepharose beads (GE Healthcare, Chicago, IL, USA) prior to adding the antibody. Next, after removing the protein A-Sepharose by centrifugation, the supernatant was incubated at 4 °C overnight with appropriate antibodies. After the addition fresh protein A-Sepharose bead, the incubation was continued for an additional one hours, and then beads were washed five times with RIPA buffer. The immune complexes were further analyzed by immunoblotting.

### 4.6. Immunofluorescence Microscopy

To visualize DNA damage foci, cells cultured on cover slips coated with poly-L-lysine (Sigma, Saint Louis, MO, USA) were irradiated at 5 Gy and allowed to recover at 37 °C for adequate times. Cells were fixed with 4% paraformaldehyde for 10 min and ice-cold 98% methanol for 5 min, followed by permeabilization with 0.3% Triton X-100 for 10 min at room temperature. After blocking using 5% BSA (Sigma), cells were single or double immunostained with primary antibodies against the indicated proteins and appropriate Alexa Fluor 488- (green, Molecular Probe, Eugene, OR, USA), Alexa Fluor 594- (red, Molecular Probe) conjugated secondary antibodies. Fluorescence images were taken using a confocal microscope (Zeiss LSM 510 Meta; Carl Zeiss, Oberkochen, Germany) and analyzed with Zeiss microscope image software ZEN (Carl Zeiss). The foci number per cells was counted at least 100 cells.

### 4.7. Laser Microirradiation

To measure accumulation of GFP-MDC1 at microirradiated genomic regions, GFP-MDC1 transfected-control, and KPNA2-depleted HeLa cells were cultured onto a 35-mm round glass dish and 10 μM 5-bromo-2’-deoxyuridine (BrdU, Sigma) was added to the medium for 24 h. DSBs were then induced by microirradiation with a 405 nm laser 2 s irradiation time (100 lines/susing an A1 confocal microscope (Nikon, Japan). Images were acquired every 1 min for 10 min.

### 4.8. Antibodies

The following antibodies were used for western blot analysis: anti-MDC1 rabbit polyclonal antibody (R2, manufactured in our lab) [1], anti-KPNA2 mouse monoclonal antibody (sc-55538, Santa cruz, Dallas, TX, USA), anti-HA mouse monoclonal antibody (sc-7392, Santa Cruz), anti-GFP mouse monoclonal antibody (sc-5286, Santa Cruz), anti-Histone3 rabbit polyclonal antibody (ab1791, Abcam, Cambridge, UK), and anti-α-Tubulin mouse monoclonal antibody (sc-5286, Santa Cruz). For immunoprecipitation assay, anti-HA rabbit polyclonal antibody (F-7, Santa Cruz), anti-GFP mouse monoclonal antibody (sc-5286, Santa Cruz), anti-MDC1 rabbit polyclonal antibody (R2, manufactured in our lab), and anti-KPNA2 mouse monoclonal antibody (sc-55538, Santa cruz) were used. The following antibodies were used for immunofluorescence staining: anti-MDC1 rabbit polyclonal antibody (R2), anti-HA mouse monoclonal antibody (sc-7392, Santa Cruz), anti-RNF8 goat polyclonal antibody (ab15850, Abcam), anti-53BP1 rabbit polyclonal antibody (sc-22760, Santa Cruz), anti-BRCA1 mouse monoclonal antibody (sc-6954, Santa cruz), anti-γH2AX mouse monoclonal antibody (05-636-1, Millipore, Burlington, MA, USA), and anti-RNF168 rabbit polyclonal antibody (ABE367, Millipore).

### 4.9. Neutral Comet Assay

Cells were left untreated or treated with 5 Gy γ-irradiation, followed by the incubation in culture medium at 37 °C for adequate times. Cells were harvested (20 μL, 1 × 10^5^ cells per pellet), mixed with 200 μL low-melting temperature agarose, and layered onto agarose-coated glass slides. The slides were maintained in the dark at 4 °C for all of the remaining steps. Slides were immerged in lysis solution (Cat. #4250-050-01, TREVIGEN® Instructions, Gaithersburg, MD, USA) for 1 h at 4 °C and then placed into a horizontal electrophoresis apparatus filled with fresh neutral electrophoresis solution (100 mM Tris, 300mM Sodium Acetate at pH 9.0) for 30 min. After electrophoresis (~30 min at 1 V/cm tank length), slides were air-dried and stained with 30–50µl of SYBR green (Lonza, Basel, Switzerland). The slides were analyzed at × 400 magnification using a fluorescence microscope (Nikon). The microscope images revealed circular shapes indicating undamaged DNA, or comet-like shapes indicating the DNA had migrated out from the head to form a tail (damaged DNA). Average comet tail moment was scored for 40–50 cells/slide using a computerized image analysis system (Komet5.5, Andor Technology, Belfast, UK).

### 4.10. Clonal Survival Assay

After treatment with irradiation, 5 × 10^2^ cells were immediately seeded on 60 mm dish in triplicate and grown for 2–3 weeks at 37 °C to allow colony formation. Colonies were stained with 2% methylene blue/50% ethanol and were counted. The fraction of surviving cells was calculated as the ratio for the plating efficiency of treated cells over untreated cells.

### 4.11. DR-GFP Assay

To measure the HR repair, U2OS-DR-GFP cells were transfected with control, KPNA2, MDC1 siRNA using lipofectamine RNAimax, and then infected with I-SceI-carrying adenovirus at an estimated MOI of 10. After 72 h, GFP-positive cells were measured by fluorescence-activated cell sorting (FACSCalibur, BD Bioscience, Franklin Lakes, NJ, USA). The acquired data was analyzed using CellQuest Pro software (BD Bioscience).

### 4.12. Statistical Analysis

Data in all experiments were represented as mean ± standard deviation (SD) for three independent experiments. Statistical comparisons were carried out using two-tailed paired Student’s t-test, where *p* < 0.01 (**) was considered statistically significant.

## Abbreviations

DDR	DNA damage response
DSBs	Double stranded breaks
FHA	Forkhead-associated
HR	Homologous recombination
KPNA2	Karyopherin α-2
MDC1	Mediator of DNA damage checkpoint 1
NLS	Nuclear localization sequence
tBRCT	two BRCA1 carboxyl-terminal
